# Pre- and Neonatal Imprinting on Immunological Homeostasis and Epithelial Barrier Integrity by *Escherichia coli* Nissle 1917 Prevents Allergic Poly-Sensitization in Mice

**DOI:** 10.3389/fimmu.2020.612775

**Published:** 2021-02-17

**Authors:** Priya J. Sarate, Dagmar Srutkova, Nora Geissler, Martin Schwarzer, Irma Schabussova, Aleksandra Inic-Kanada, Hana Kozakova, Ursula Wiedermann

**Affiliations:** ^1^Institute of Specific Prophylaxis and Tropical Medicine, Center for Pathophysiology, Infectiology and Immunology, Medical University of Vienna, Vienna, Austria; ^2^Laboratory of Gnotobiology, Institute of Microbiology of the Czech Academy of Sciences, Novy Hradek, Czechia

**Keywords:** allergic poly-sensitization, germ-free, BALB/c, mucosal tolerance, mouse model, *Escherichia coli* Nissle 1917, hygiene hypothesis

## Abstract

A steady rise in the number of poly-sensitized patients has increased the demand for effective prophylactic strategies against multi-sensitivities. Probiotic bacteria have been successfully used in clinics and experimental models to prevent allergic mono-sensitization. In the present study, we have investigated whether probiotic bacteria could prevent poly-sensitization by imprinting on the immune system early in life. We used two recombinant variants of probiotic *Escherichia coli* Nissle 1917 (EcN): i) EcN expressing birch and grass pollen, poly-allergen chimera construct (EcN-Chim), and ii) an “empty” EcN without allergen expression (EcN-Ctrl). Conventional mice (CV) were treated with either EcN-Chim or EcN-Ctrl in the last week of the gestation and lactation period. Gnotobiotic mice received one oral dose of either EcN-Chim or EcN-Ctrl before mating. The offspring from both models underwent systemic allergic poly-sensitization and intranasal challenge with recombinant birch and grass pollen allergens (rBet v 1, rPhl p 1, and rPhl p 5). In the CV setting, the colonization of offspring *via* treatment of mothers reduced allergic airway inflammation (AAI) in offspring compared to poly-sensitized controls. Similarly, in a gnotobiotic model, AAI was reduced in EcN-Chim and EcN-Ctrl mono-colonized offspring. However, allergy prevention was more pronounced in the EcN-Ctrl mono-colonized offspring as compared to EcN-Chim. Mono-colonization with EcN-Ctrl was associated with a shift toward mixed Th1/Treg immune responses, increased expression of TLR2 and TLR4 in the lung, and maintained levels of zonulin-1 in lung epithelial cells as compared to GF poly-sensitized and EcN-Chim mono-colonized mice. This study is the first one to establish the model of allergic poly-sensitization in gnotobiotic mice. Using two different settings, gnotobiotic and conventional mice, we demonstrated that an early life intervention with the EcN without expressing an allergen is a powerful strategy to prevent poly-sensitization later in life.

## Introduction

The prevalence of allergic poly-sensitization in children has increased significantly over the last decades (1–6). Nowadays, it is approximated that 14 to 45% of children suffer from poly-sensitization (3, 5, 6), which starts as early mono-sensitization to aeroallergens, such as birch and grass pollen allergens (4, 7–11). Despite the availabilities of conventional allergen-specific immunotherapy, based on the injection of increasing doses of allergen mixtures, the exposure to these allergens could pose a risk for the development of new sensitizations. There is a pressing need for the development of novel prophylactic therapies (12–16); therefore, the induction of immune tolerance early in life could be an excellent strategy for preventing poly-sensitization in children.

The priming of the immune system is particularly effective during the early period of life (17). Early life stages such as prenatal, perinatal, and early-postnatal period are crucial for establishing balanced gut microflora, which has been associated with reduced allergy development (18–22). The importance of immunological imprinting has been shown by clinical intervention studies, where early probiotic applications to mothers prevented allergies in their children (23–25). Using a mouse model of mono-sensitization to the major birch pollen allergen Bet v 1, we have previously shown that the perinatal and neonatal colonization with particular wild type probiotic bacteria (21, 26) or with recombinant probiotic bacteria expressing Bet v 1 (22) during the gestation and lactation period, reduced the development of Bet v 1-specific allergy.

Here, we investigated whether *Escherichia coli* Nissle 1917 (EcN), a probiotic Gram-negative bacterial strain with strong immunomodulatory properties (27, 28), can prevent the development of poly-sensitization in mice when applied early in life. Recently, we engineered a recombinant EcN expressing either the major birch (Bet v 1) and grass (Phl p 1 and Phl p 5) (EcN-Chim) pollen allergens or EcN harboring the empty plasmid (EcN-Ctrl) (7, 28, 29) and showed that the intranasal pretreatment with EcN-Chim but not EcN-Ctrl reduced allergic poly-sensitization in adult mice (28).

In the current study, performed in conventional (CV) and monoxenic mice, we had assessed the impact of EcN-Chim and EcN-Ctrl (antigen-specific vs. non-antigen-specific tolerance induction) treatment on the experimental poly-sensitization model when probiotics were applied in the early phase of life. In this phase of life, it was of interest to investigate whether EcN bacteria itself can prevent allergy or if the expression of the respective allergen by EcN is needed for successful allergy prevention.

Therefore, our first aim was to investigate the effects of EcN-Chim and EcN-Ctrl applied orally to mothers during the last week of gestation and lactation on the development of allergy in their offspring in CV conditions. Secondly, we have used the gnotobiotic mouse model to enable mother-to-offspring early bacterial mono-colonization to understand better the EcN-Chim and EcN-Ctrl impact on the prevention of poly-sensitization.

## Material and Methods

### Bacteria and Growth Conditions

Two recombinant strains EcN previously described (28) were used in the current study: i) EcN-Chim: a clone expressing multi-allergen chimer Phl p 5-Bet v 1-Phl p 1 along with fluorescent mCherry; ii) EcN-Ctrl clone expressing only mCherry without allergen. Both recombinant clones were grown and selected on Luria-Bertani (LB) agar plates with 20 µg ml^−1^ chloramphenicol (CM) at 37°C overnight, as described in Sarate et al., 2019 (28). In liquid medium, recombinant EcN-Chim and EcN-Ctrl strains were cultivated at 30°C on 200 RPM overnight in LB-medium containing 20 µg/ml chloramphenicol (28).

### Antigens

Recombinant (r) allergens Bet v 1, Phl p 1, and Phl p 5 were purchased from Biomay GmbH (Vienna, Austria).

### Animals and Ethics Statement

For perinatal study, pregnant conventional BALB/c mice in the last week of gestation were purchased from Charles River (Sulzfeld, Germany). The animals were maintained under controlled conventional housing conditions and provided with standard diet and water *ad libitum*. All experiments were approved by the Animal Experimentation Committee of the Medical University of Vienna and by the Federal Ministry of Science and Research (BMWF-66.009/0384-WF/V/3b/2015).

For germ-free (GF) mice studies in neonates, germ-free female BALB/c mice were kept under sterile conditions in Trexler-type plastic isolators, and the absence of aerobic and anaerobic bacteria, molds, and yeast was confirmed every two-weeks by standard microbiological methodology (31, 32). The mice were kept in a room with a 12 h light-dark cycle at 22°C, fed an irradiated sterile diet (Altromin 1414, Altromin, Germany), and provided sterile autoclaved water *ad libitum*. The animal experiments were approved by the Committee for the Protection and Use of Experimental Animals of the Institute of Microbiology of the Czech Academy of Sciences (approval ID: 23/2018).

### Mouse Model of Poly-Sensitization Using Recombinant Birch and Grass Pollen Allergens

Poly-sensitization was done as described previously (28). Briefly, female BALB/c mice were sensitized with three intraperitoneal injections (days 10, 24, and 39) of a mixture of 5 µg rBet v 1, 5 µg rPhl p 1, and 5 µg rPhl p 5 adsorbed to aluminum hydroxide (Al(OH)_3_; Serva, Heidelberg, Germany) (0.68 mg/ml). To induce airway inflammation, one week after the last intraperitoneal immunization, mice were anesthetized by 2% isoflurane in an anesthetic induction chamber and challenged intranasally with 30 µl of a mixture of 5 µg rBet v 1, 5 µg rPhl p 1, and 5 µg rPhl p 5 for 3 consecutive days.

Mice were sacrificed 72 h after the last challenge by exposure to carbon dioxide and blood was collected from the facial vein of mice at the end of the experiment. Sera were collected and stored at −20°C until used.

### The Perinatal Approach in Conventional Mice

Recombinant EcN-Chim and EcN-Ctrl strains were cultivated at 200 RPM, 30°C overnight in LB medium. Bacterial cells were collected and washed with ice-cold PBS. For perinatal application, bacterial cultures were adjusted to 1 x 10^9^ CFU/300µl of gavage buffer (0.2 M NaHCO_3_ buffer containing 1% glucose, pH 8) per mice. Pregnant mice in the last week of gestation were pretreated orally with either EcN-Chim or EcN-Ctrl during the gestation (every day) and lactation (every second day). On day 21, female offspring (n = 6 to 9 mice per group) derived from these mothers were separated and sensitized intraperitoneally with 5 µg rBet v 1, 5 µg rPhl p 1, and 5 µg rPhl p 5 followed by challenge with 5 µg rBet v 1, 5 µg rPhl p 1, and 5 µg rPhl p 5 as described in the poly-sensitization model (21). Mice were sacrificed 72 h after the last challenge, bronchoalveolar lavage (BAL), lung samples and blood were collected for further analysis. After centrifugation, sera were collected and stored at −20°C for further analysis.

### The Perinatal Approach in GF Mice

Recombinant EcN-Chim and EcN-Ctrl strains were prepared as described above (in a sterile condition) for the mono-colonization in GF mice. Eight-week-old GF mice were colonized with a single dose (2 × 10^8^ CFU/200 µl PBS), either EcN-Chim or EcN-Ctrl by intragastric gavage and mated 10 days later. During the experiment, drinking water was supplemented with chloramphenicol (200 mg/L) to ensure the long‐term stability of the recombinant EcN strains *in vivo*. The stability of colonization was checked by plating of feces on LB agar and counting after aerobic cultivation for 24 h at 37°C. Colonization remained stable throughout the experiment and reached levels of 0.8-1.2 x 10^10^ CFU/g feces (EcN-Chim) and 0.7-1.4 x 10^10^ CFU/g feces (EcN-Ctrl).

On day 21, neonatally colonized female offspring either by EcN-Chim or EcN-Ctrl, as well as germ-free controls, were separated from their mothers and divided into two groups. One group was sensitized intraperitoneally with 5 µg rBet v 1, 5 µg rPhl p 1, and 5 µg rPhl p 5 (Biomay, Austria) emulsified in 100 μl of Al(OH)_3_ (Serva, Germany) three times at a 14-day intervals. The other group was colonized but not sensitized and challenged. One week after the last i.p. immunization, mice were anesthetized by isoflurane and challenged intranasally with 30 μl of the mixture of 5 μg rBet v1, 5 μg rPhl p 1, and 5 μg rPhl p 5 for three consecutive days as described in the poly-sensitized model. Mice were sacrificed 72 h after the last challenge, and blood, BAL and lung samples were collected for further analysis.

### Characterization of Airway Inflammation and Allergic Poly-Sensitization

#### BAL

To evaluate the allergic airway inflammation (AAI), mice were terminally anesthetized, the tracheas were cannulated, and lungs were lavaged with 2 x 0.5 ml PBS (30, 33). BAL fluids were centrifuged at 300 x g for 5 min at 4°C and cell-free supernatants were stored at −20°C for further analysis. Cell pellets were recovered for cellular analysis. After counting, cytospins were prepared by spinning cells onto microscope slides (Shandon Cytospin^®^, Shandon Southern Instruments, USA) and staining with H&E (Hemacolor^®^, Merck, Darmstadt, Germany). Cytospin preparations were differentiated according to standard morphologic criteria by counting 200 cells *via* light microscopy. Collected supernatants were then analyzed for IL-5 and IL-13 response in BAL fluids. Macrophages, lymphocytes, eosinophils, and neutrophils per slide were counted under the light microscope (Nikon Eclipse; 100x magnification) (200 cells per count). Results represent the absolute numbers of cells.

#### Lung Histology

Small lung tissues were excised and fixed with 7.5% formaldehyde-PBS, followed by paraffin-embedding. Lung sections (5 µm thick) were stained with periodic acid-Schiff (PAS) stain. Lung histological pathology was evaluated using light microscopy. Numbers of PAS-positive, mucus-producing Goblet cells in the bronchial epithelium were counted by an investigator blinded to the experimental setting. Results are given as the mean number of goblet cells per millimeter of the basement membrane (34).

#### Allergen-Specific Antibodies in Serum

Allergen-specific antibody levels in mouse sera (IgE, IgG1, and IgG2a) were determined by ELISA as previously described (35). Briefly, microtiter plates (Nunc, Roskilde, Denmark) were coated with each of the recombinant allergens Bet v 1, Phl p 1, or Phl p 5 (2 μg/ml) and incubated with mouse sera. Antibody detection was performed using rat anti-mouse IgE, IgG1, IgG2a, followed by peroxidase-conjugated mouse anti-rat IgG. Results show the optical density (OD) values after subtraction of baseline levels from pre-immune sera.

The determination of the allergenic antibody serum activity was performed as previously described (30). Briefly, RBL-2H3 cells were passively sensitized by incubation with serum samples of the respective experimental groups and their degranulation was induced by addition of recombinant allergens rBet v 1, rPhl p 1 and rPhl p 5 (0.3µg/ml) diluted in Thyrode's buffer. Supernatants were analysed for β-hexosaminidase activity. Results are reported as percentages of total β-hexosaminidase release after adding 1% Triton X-100 and are shown after subtraction of baseline release levels obtained with pre-immune sera.

#### Allergen-Specific Cytokine Detection in BAL

BAL supernatants collected during sacrifice were analyzed for IL-5 and IL-13 levels by using an ELISA specific for murine cytokines (Ready-Set-Go ELISA Kit eBioscience, USA) according to the manufacturer’s instructions.

#### Quantification of mRNA Expression by Real-Time (RT)-PCR

Total RNA was extracted from lung samples of mice from all treatment and controls group at the end of the experiment. RNA quantification was performed using ND-1000 Spectrophotometer (Nanodrop Technologies Inc., Wilmington, USA) and cDNA was obtained using reverse-transcriptase kit (BIO-RAD, Vienna, Austria). Expression of IL-10, Foxp3, and IFNγ mRNA was measured by RT-PCR as described previously (30). The housekeeping gene Glyceraldehyde 3-phosphate dehydrogenase (GAPDH) and β-actin (Universal Probe Library probe #64; Roche) were used as a control to standardize the amount of sample cDNA. Data are presented as the ratio of the target genes expression to GAPDH and β-actin expression.

#### Immunohistochemical Identification of TLR2, TLR4, and Zonulin-1 (ZO-1) in Lung Tissue of Mono-Colonized Mice

For TLR2 and TLR4 analysis, the 3 μm lung sections were deparaffinized and antigens were retrieved in 0.01 M citrate buffer (pH 6) using a microwave vessel for 10 min. After cooling down and washing by PBS, endogenous peroxidase was blocked with 3% hydrogen peroxide in PBS for 15 min. Non-specific adsorption was eliminated by incubation of the sections in 10% normal rabbit serum in PBS for 1 h. Samples were incubated overnight with polyclonal goat anti-TLR2 (4 μg/ml) or anti-TLR4 (4 μg/ml) (Santa Cruz Biotechnology, Dallas, Texas, USA) at 4°C. After washing in PBS, sections were incubated with rabbit anti-goat IgG conjugated with horseradish peroxidase (1:200 in PBS) (Jackson, ImmunoLabs., West Grove, PA, USA) for 1 h and stained with DAB (3,3′-Diaminobenzidine) (Dako, Carpinteria, CA, USA) for 3 min. The counterstain was carried out with hematoxylin, samples were mounted by the Paramount Aqueous Mounting medium (Dako, Carpinteria, CA, USA) and viewed under an Olympus BX 40 microscope with 40x objective, equipped with and Olympus DP 70 digital camera.

ZO-1 analysis was done as previously described (36). Briefly, the 3 μm lung sections were deparaffinized, antigens were retrieved by protease from *Streptomyces griseus* (1 mg/ml, type XIV, Sigma-Aldrich, St. Luis, MO, USA) at 37°C for 8 min, washed with PBS and endogenous peroxidase was blocked with 3% hydrogen peroxide in PBS for 15 min. Non-specific adsorption was eliminated by incubation of the sections in 10% normal goat serum in PBS for 1 h. Samples were incubated overnight with polyclonal rabbit anti-ZO-1 (5 μg/ml, Santa Cruz Biotechnology, Dallas, Texas, USA) at 4°C. After washing in PBS, sections were incubated with goat anti-rabbit IgG conjugated with horseradish peroxidase (1:200 in PBS) (Jackson, ImmunoLabs., West Grove, PA, USA) for 1 h and stained with DAB (3,3′-Diaminobenzidine) (Dako, Carpinteria, CA, USA) for 1 min.

Quantification of TLR2, TLR4 and ZO-1 immunohistochemistry was done by measuring the optical density (OD). The OD analysis of the TLR4, TLR2 and Zonulin-1 images was evaluated using ImageJ (Fijiv2.0.0; National Institutes of Health, Bethesda, MD). Briefly, 8-bit RGB DAB-stained images were processed through color deconvolution (37), and threshold settings and mean gray value was assessed in the selected area of bronchus/bronchiolar epithelial layer in DAB-extracted images. OD was estimated by the following formula: OD = log (max intensity/mean gray value intensity), where max intensity = 255 (38). OD of background (field without any tissue) was subtracted from OD of epithelial cell layer. Results from two to five bronchi/bronchiole from individual mouse were pooled and five mice per each group were evaluated (37, 38).

### Statistical Analysis

Statistical analysis was conducted using GraphPrism, ver. 6. For comparison of more groups the One-Way ANOVA was applied followed by the Bonferroni’s Multiple Comparison Test unless otherwise specified. All data are shown as mean ± SEM. Significant differences were considered at P < 0.05 (*), P < 0.01 (**), P < 0.001 (***).

## Results

### Conventional Mouse Model

#### Offspring of Mothers Treated With EcN-Ctrl Exhibited Reduced Allergic Airway Inflammation (AAI)

**Figure 1:**
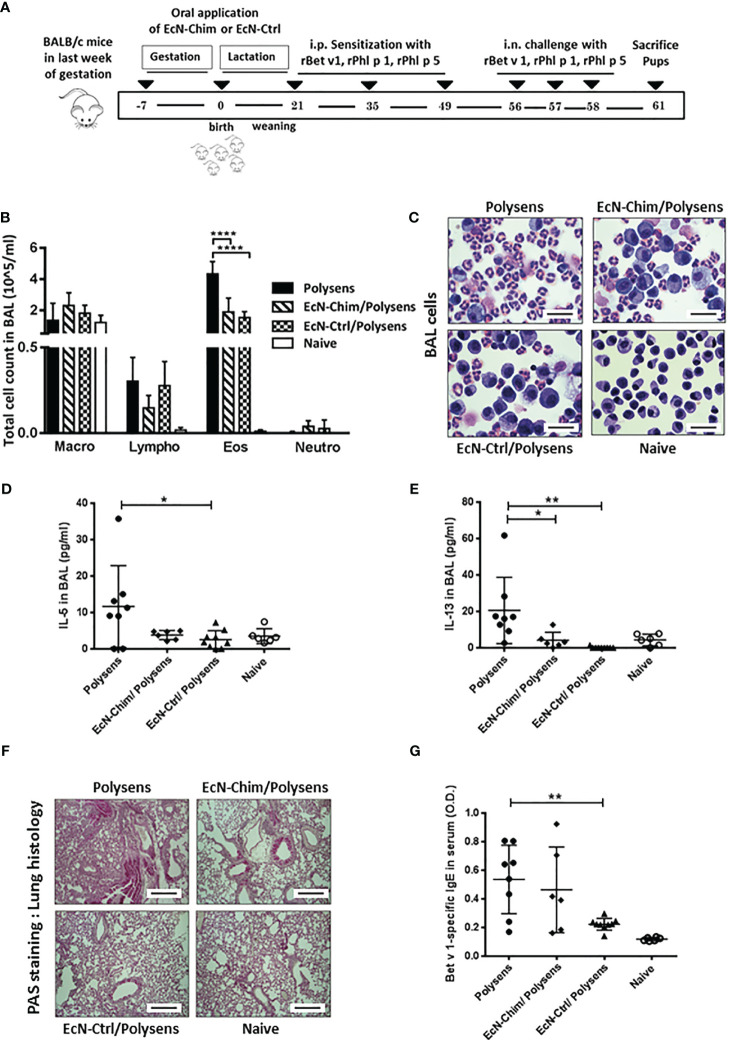
Perinatal application of *Escherichia coli* Nissle 1917 (EcN)-Chim and EcN-Ctrl in conventional mice reduced airway inflammation. **(A)** Schematic representation of the perinatal application of recombinant EcN expressing birch-grass pollen chimera (EcN-Chim) and EcN expressing empty plasmid (EcN-Ctrl) in a conventional mouse model of poly-sensitization. Female BALB/c mice were given either EcN-Chim or EcN-Ctrl orally during the gestation and lactation. On day 21, female offspring derived from these mothers were separated and were then sensitized and challenged with 5 µg Bet v 1, 5 µg Phl p 1, and 5 µg Phl p 5 as described in poly-sensitization model. Mice were sacrificed 72 h after the last challenge. **(B)** Absolute numbers of macrophages, lymphocytes, eosinophils, and neutrophils in bronchoalveolar lavage (BAL) and **(C)** Representative cytospins of BAL of one mouse per group stained with hematoxylin and eosin (H&E; 100x magnification). **(D)** IL-5 and **(E)** IL-13 cytokines in BAL. **(F)** Representative lung tissue sections of one mouse per group stained with Periodic Acid Schiff (PAS) (Red; 10 x magnification; scale bars 100 µm). **(G)** Levels of Bet v 1-specific serum IgE. **(B, D, E, G)** represents the mean ± SEM from two experiments (total n = 6 to 9 mice per group). Error bars show mean ± SEM. *P < 0.05, **P < 0.01, ****P < 0.0001 by the One-way ANOVA followed by the Bonferroni’s Multiple Comparison Test.

To assess the effects of perinatal interventions with probiotic bacteria on poly-sensitization, mice were treated orally with either EcN-Chim or EcN-Ctrl during the gestation and lactation period, and their offspring were sensitized and challenged with rBet v 1, rPhl p 1, and rPhl p 5 (**Figure 1A**). The offspring colonized perinatally with EcN-Ctrl exhibited reduction in BAL eosinophils (P < 0.001), IL-5 (P < 0.05), and IL-13 (P < 0.01) levels as compared to poly-sensitized offspring (**Figures 1B–E**). Offspring colonized with EcN-Chim exhibited reduced IL-13 (P < 0.05) as well as eosinophil level (P < 0.05) in BAL compared to poly-sensitized animals. Lung histology revealed reduced mucus production in the lung of offspring colonized with either EcN-Chim or EcN-Ctrl as compared to poly-sensitized controls (**Figure 1F**). Besides, perinatal colonization with EcN-Ctrl induced a reduction in Bet v 1-specific serum IgE as compared to poly-sensitized controls (P < 0.01) (**Figure 1G**).

### Gnotobiotic Mouse Model

#### Perinatal and Neonatal Mono-Colonization With Either EcN-Chim or EcN-Ctrl Prevented AAI

**Figure 2:**
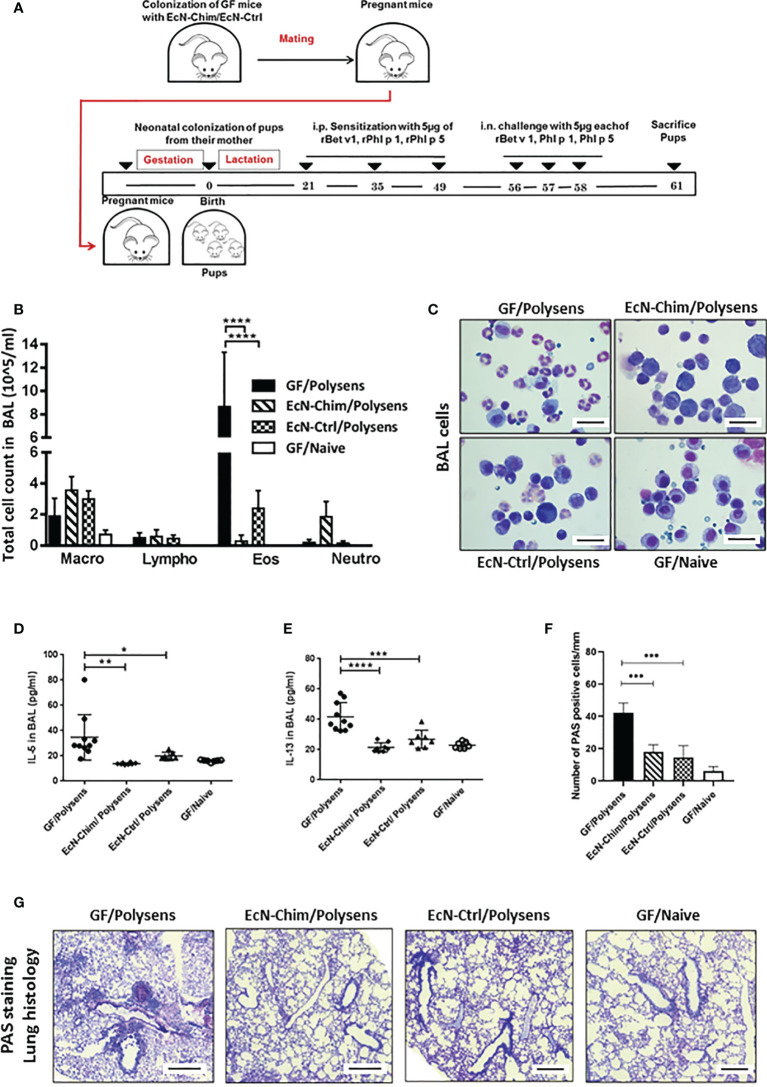
Perinatal and neonatal mono-colonization of *Escherichia coli* Nissle 1917 (EcN)-Chim and EcN-Ctrl in germ-free (GF) mice reduced airway inflammation. **(A)** Schematic representation of the neonatal colonization of GF mice with recombinant EcN expressing EcN-Chim and EcN-Ctrl in a gnotobiotic mouse model of poly-sensitization. Eight-week-old GF mice were colonized with a single dose of either EcN-Chim or EcN-Ctrl by intragastric gavage and mated 10 days later. On day 21, all mono-colonized female offspring were separated from their mothers and divided into two groups. One group was sensitized and challenged with 5 µg rBet v 1, 5 µg rPhl p 1, and 5 µg rPhl p 5 and other not. Mice were sacrificed 72 h after the last challenge **(B)** Absolute numbers of macrophages, lymphocytes, eosinophils, and neutrophils in bronchoalveolar lavage (BAL). **(C)** Representative cytospins of BAL of one mouse per group stained with H&E 100x magnification). **(D)** IL-5 and **(E)** IL-13 cytokines in BAL. **(F)** Quantification of mucus-producing goblet cells. **(G)** Representative lung tissue sections of one mouse per group stained with Periodic Acid Schiff (PAS) (Red; 10x magnification; scale bars 100 µm); arrows indicate cell infiltration. **(B, D–F)** represents mean ± SEM from two experiments (total n = 7 to 10 mice per group). Error bars show mean ± SEM. *P < 0.05, **P < 0.01, ***P < 0.001, ****P < 0.0001 by the One-way ANOVA followed by the Bonferroni’s Multiple Comparison Test.

In a gnotobiotic mouse model, perinatal/neonatal mono-colonization of offspring *via* their mothers with either EcN-Chim or EcN-Ctrl (**Figure 2A**) significantly reduced AAI as compared to GF poly-sensitized controls (**Figures 2B–F**). This was reflected by reduced recruitment of eosinophils (EcN-Chim, P < 0.0001; EcN-Ctrl, P < 0.001), as well levels of IL-5 (EcN-Chim, P < 0.01; EcN-Ctrl, P< 0.05), and IL-13 (EcN-Chim, P < 0.0001; EcN-Ctrl, P < 0.001) in BAL samples compared to GF poly-sensitized controls (**Figures 2B–E**). Histological analysis revealed reduced mucus production by airway-lining goblet cells in the lungs of the groups colonized with either EcN-Chim (P < 0.0001) or EcN-Ctrl (P < 0.0001) compared to GF poly-sensitized control (**Figures 2F, G**).

#### Perinatal and Neonatal Mono-Colonization With Either EcN-Chim or EcN-Ctrl Inhibits the Development of Th2-Type Allergen-Specific Immune Response

**Figure 3:**
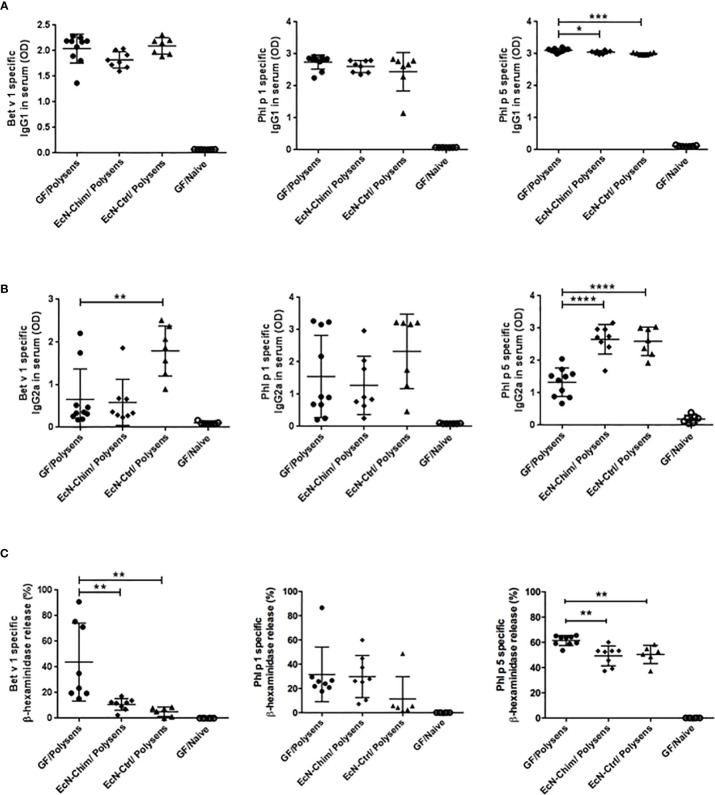
Perinatal and neonatal mono-colonization with *Escherichia coli* Nissle 1917 (EcN)-Chim and EcN-Ctrl inhibits the development of Th2-type allergen-specific immune response. Mice were treated as indicated in **Figure 2A**. Serum samples were obtained from mice on sacrifice day. Allergen-specific antibody levels in mouse sera were determined by ELISA and RBL. The figure represents levels of Bet v 1, Phl p 1, Phl p 5-specific serum **(A)** IgG1 **(B)** IgG2a, and **(C)** IgE. Data represent mean ± SEM from two experiments (total n = 7 to 10 mice per group). Error bars show mean ± SEM. *P < 0.05, **P < 0.01, ***P < 0.001, ****P < 0.0001 by the One-way ANOVA followed by the Bonferroni’s Multiple Comparison Test.

Mono-colonization with EcN-Chim and EcN-Ctrl significantly reduced Phl p 5-specific IgG1 levels (EcN-Chim, P < 0.05; EcN-Ctrl, P < 0.001) in sera (**Figure 3A**). Perinatal/neonatal mono-colonization with EcN-Ctrl led to a substantial increase of Bet v 1 (P < 0.01) and Phl p 5-specific (P < 0.0001) IgG2a antibody levels in serum in comparison with GF poly-sensitized control mice (**Figure 3B**). Mono-colonization with EcN-Chim led to increased IgG2a against Phl p 5 (P < 0.0001) compared to GF poly-sensitized control (**Figure 3B**). By measuring the IgE-dependent basophil degranulation, we have shown that cells derived from EcN-Chim and EcN-Ctrl-colonized mice and stimulated with rBet v 1 (P < 0.01) and rPhl p 5 (P < 0.01) exhibited reduced antigen-specific β-hexosaminidase release compared to the sera of GF poly-sensitized controls (**Figure 3C**). No significant difference was observed for Phl p 1-specific IgG2a, IgG1, and β-hexosaminidase release in EcN-Chim and EcN-Ctrl mice in comparison with GF poly-sensitized controls (**Figures 3A–C**).

#### Perinatal and Neonatal Mono-Colonization With EcN-Ctrl but Not EcN-Chim Induced IL-10, Foxp3, and IFNγ mRNA Expression in the Lung

**Figure 4:**
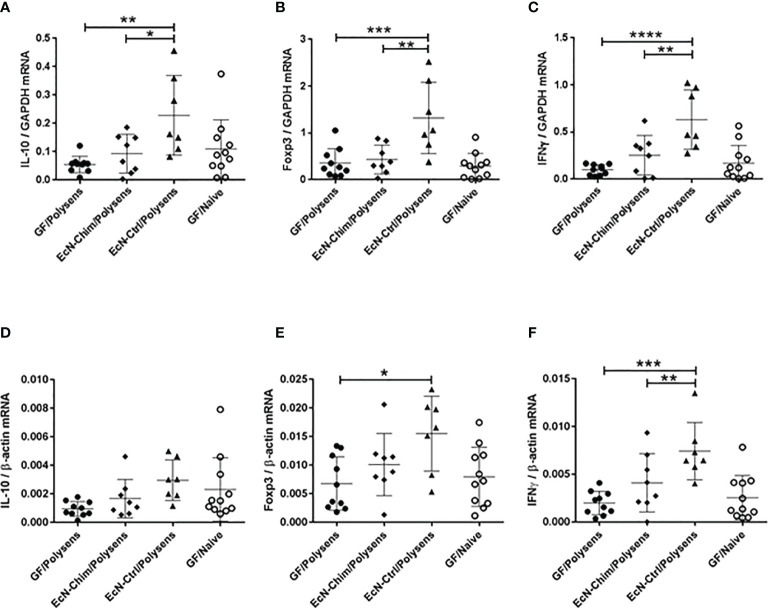
Perinatal and neonatal mono-colonization with *Escherichia coli* Nissle 1917 (EcN)-Ctrl led to increased IL-10, Foxp3, and IFNγ mRNA expression in the lung. Mice were treated as indicated in **Figure 2A**. After sacrifice, lung samples were collected and expression of IL-10, Foxp3 and IFNγ mRNA was measured by real-time (RT)-PCR. The figure represents the ratio of the target genes: **(A)** IL-10, **(B)** Foxp3, and **(C)** IFNγ to the GAPDH reference gene and **(D)** IL-10, **(E)** Foxp3, and **(F)** IFNγ to the β-actin reference gene. Data represents mean ± SEM from two experiments (total n = 7 to 10 mice per group) analyzed by the One-way ANOVA followed by the Bonferroni’s Multiple Comparison Test. Error bars show mean ± SEM. Data represents mean ± SEM from two experiments (total n = 7 to 10 mice per group). Error bars show mean ± SEM. *P < 0.05, **P < 0.01, ***P < 0.001, ****P < 0.0001 by the One-way ANOVA followed by the Bonferroni’s Multiple Comparison Test.

Expression of IL-10, Foxp3, and IFNγ mRNA in lungs were analyzed with two reference genes GAPDH (**Figures 4A–C**) and β-actin (**Figures 4D–F**). Although mono-colonization with both EcN-Ctrl and EcN-Chim reduced allergy in poly-sensitized mice, only the mono-colonization with EcN-Ctrl triggered the increased expression of IL-10 (P < 0.01), Foxp3 (P < 0.001), and IFNγ (P < 0.0001) mRNA in the lung as compared to GF poly-sensitized control (**Figures 4A–C, E, F**). Mono-colonization with EcN-Chim did not influence the levels of IL-10, Foxp3, and IFNγ mRNA expression in the lung in comparison with GF poly-sensitized mice. EcN-Ctrl showed significantly higher expression of IL-10 (P < 0.05), Foxp3 (P < 0.01), and IFNγ (P < 0.01) compared to EcN-Chim (**Figures 4A–C, F**).

#### Perinatal and Neonatal Mono-Colonization With EcN-Chim and EcN-Ctrl Increased Expression of TLR2 and TLR4 as Well as Maintained Epithelial Barrier Integrity in the Lung by ZO-1 Expression

**Figure 5:**
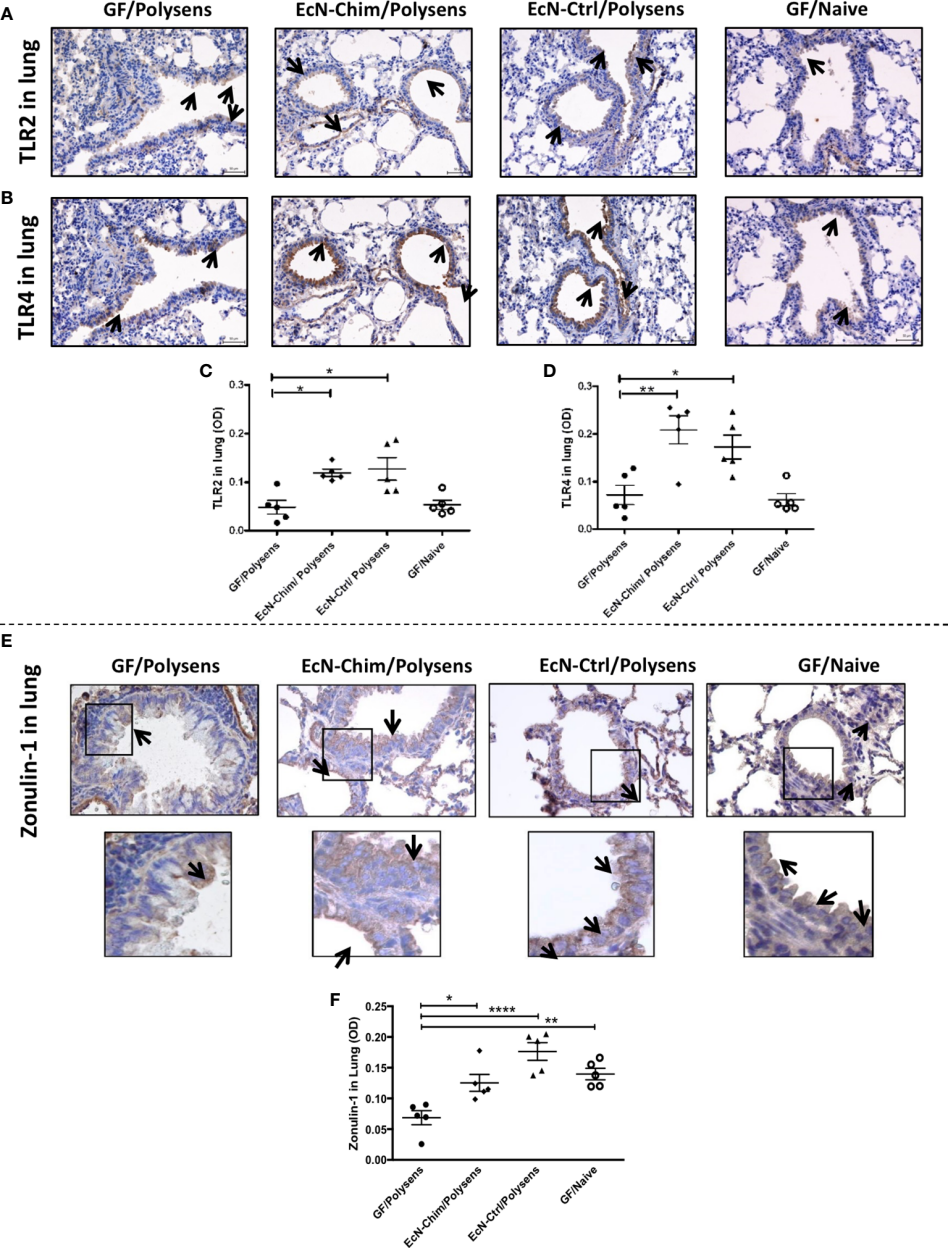
Perinatal and neonatal mono-colonization of either EcN-Chim or EcN-Ctrl activated both TLR2 and TLR4 expression and maintained ZO-1 levels in the lung. Mice were treated as indicated in **Figure 2A**. For immunohistochemistry analysis, lung sections were processed and stained as described in Material and Methods using antibodies against TLR2, TLR4, and ZO-1 followed by 3,3′-Diaminobenzidine (DAB) staining. Samples were analyzed under a light microscope with a 40x objective. Immunohistochemistry staining for TLR2 **(A)**, TLR4 **(B)**, and ZO-1 **(E)** is represented by a brown color (indicated by arrows). Quantification of TLR2 **(C)**, TLR4 **(D)**, and ZO-1 **(F)** expression was performed by optical density analysis (OD) of bronchial epithelial cell layer using ImageJ software. **(C, D, F)** represents mean ± SEM from two experiments (five mice per group were tested). Error bars show mean ± SEM. *P < 0.05, **P < 0.01, ****P < 0.0001 by the One-way ANOVA followed by the Bonferroni’s Multiple Comparison Test.

Immunochemical staining of the lungs showed increased TLR2 (**Figure 5A**) and TLR4 (**Figure 5B**) expression in the lungs of mono-colonized mice. This increase was confirmed by quantification of TLR2 and TLR4 using ImageJ software. Quantification data showed moderate increase in TLR2 (**Figure 5C**) and strong stimulation of TLR4 (**Figure 5D**) expression in the lung of mice mono-colonized with either EcN-Chim or EcN-Ctrl compared to and poly-sensitized controls.

Lung immunohistochemistry showed maintained levels of ZO-1 in bronchial epithelial cells, in both EcN-Chim and EcN-Ctrl mono-colonized mice as compared to GF polysensitized mice (**Figure 5E**). Quantification of ZO-1 showed significant decrease in ZO-1 in GF polysensitized mice as compared to both mono-colonized mice (**Figure 5F**). An apparent reduction in epithelial ZO-1 levels was observed in GF poly-sensitized controls as compared to GF naïve group (**Figure 5F**).

## Discussion

The constant increase in birch and grass pollen poly-sensitization and the lack of appropriate therapy are raising a considerable demand for a robust prophylactic intervention (5, 6, 39–41). It has been suggested that the immune response generated during the early phase of life to various environmental factors and antigens may be decisive for the immune response to different allergic diseases later in life (19, 20, 42–44). In this study, we have demonstrated that the perinatal and neonatal mono-colonization of mice with EcN expressing either birch-grass pollen multi-allergen, EcN-Chim, or an empty vector, EcN-Ctrl, prevented the development of poly-sensitization.

Evidence-based studies have revealed that interventions with probiotic bacteria prevent the development of allergy (45, 46). EcN is a probiotic strain with strong immunomodulatory properties and has also been used as an antigen delivery system (47–50). We have previously shown that the EcN-Ctrl was not potent enough to reduce allergic multi-sensitivities in fully-grown mice with established microflora. Therefore, we constructed a recombinant EcN strain, expressing birch and grass pollen allergens chimera, EcN-Chim, which prevented allergen-specific multi-sensitivities in adult mice after intranasal application (28). In the current study, by performing experiments in the perinatal and neonatal phase and taking advantage of early interventions, we have demonstrated that mothers-to-offspring mono-colonization not only with the EcN-Chim but also EcN-Ctrl strain, which does not express the tolerizing allergens, was sufficient to prevent allergy development.

It has been shown that various factors such as reduced infections, delivery by Cesarean section, antibiotic treatment, and microbial dysbiosis during the early age of life can influence the development of the immune system leading to an increased risk of allergic multi-sensitization, particularly in infancy (19, 51–55). The maturation of human immune system occurs early in life in parallel to the establishment of the gut microflora, i.e., approximately from the conception to the end of the second year of childhood (56, 57). Numerous clinical mono-sensitization studies have demonstrated that the early phase of life represents a “window of opportunity” where the immune system can be modulated by the application of certain probiotic bacteria (23–25, 56). In line with other studies, we have previously shown that early intervention with probiotic bacterial strains can prevent birch pollen allergy in mice (21, 22). In the current study, we have demonstrated that the immune system can be modulated by using allergen-specific and allergen-non-specific immunomodulators during the gestation and lactation period to prevent the development of poly-sensitization.

Our group has shown that the route of probiotic application is essential for the outcome of probiotic interventions in animal models of mono- and poly-sensitization (28, 35, 58). In conventional adult mice with fully established gut microflora, we have previously demonstrated that the intranasal route possesses advantages over the oral route of application in reaching the beneficial effects of treatment (28). In the current study in neonates, a single oral application of probiotic bacteria in germ-free mothers was sufficient to induce a local as well as systemic protective immune response against poly-sensitization. This finding has tremendous practical implications since the oral route of application is more convenient and the preferred route for the induction of mucosal tolerance in children against various food- and airborne-triggered allergic diseases (44, 59–61). The oral application to mothers led to the colonization of either EcN-Chim or EcN-Ctrl in offspring (data are not shown). Despite having rich commensal microflora, the EcN strain managed to colonize in the guts of CV offspring during the neonatal period and had a pronounced effect on poly-sensitization. However, it is still unclear how the colonization establishes in offspring. We can assume that this colonization occurs via contact with mothers feces and/or feeding on mothers milk. Several clinical studies demonstrated colonization with probiotic bacteria from mother to offspring *via* breast milk (62, 63).

To investigate the mechanism of tolerance induction by the EcN-Chim and EcN-Ctrl strains against poly-sensitization, we took advantage of a gnotobiotic mouse model. We found that both strains reduced allergic multi-sensitization and AAI. Mice mono-colonized with EcN-Ctrl exhibited increased Th1/Treg responses in the lung. The shift toward Th1/Treg cellular responses in the EcN-Ctrl group was associated with reduced Bet v 1- and Phl p 5-specific IgE measured by rat basophil leukemia (RBL) cells degranulation assay as the level of β-hexaminidase, and increased levels of allergen-specific IgG2a. We did not observe any difference in Phl p 1-specific β-hexaminidase release and IgG2a levels, which might be associated with the high allergenic properties of Phl p 1 (64–66). These results are in line with previous studies suggesting that anti-allergic properties of the EcN are related to the induction of specific Th1/Treg response *via* the induction of Foxp3, IL-10, and IgG2a (28, 50, 67). It is known that Foxp3 and IL-10 play a key role in allergy suppression and maintaining immune tolerance (68–70). Our data indicate that perinatal and neonatal mono-colonization with the EcN-Ctrl precludes poly-sensitization development by polarizing the Th1/Treg response in the lung.

It has been suggested that mucosal TLR2 and TLR4 orchestrate the tolerance in the gastrointestinal and respiratory tract (50, 67, 71). It was further demonstrated that the amelioration of dextran sulfate sodium-induced colitis by EcN is mediated *via* TLR-2- and TLR-4-dependent pathways (72, 73). In our current study, the lung from mice mono-colonized with the EcN-Chim and EcN-Ctrl showed increased expression of both TLR2 and TLR4 compared with GF poly-sensitized and GF naive controls. These findings support our previous *in vitro* data, demonstrating that the EcN can activate both TLR2 and TLR4 expressed in HEK293 cells (28).

Apart from being an important site to initiate the immune response, airway epithelial mucosa plays a vital role in protecting the body from the environment and maintaining the inner homeostasis in the lung (74, 75). Often in allergy, the structure and functions of airway epithelial barriers are markedly impaired by the lung inflammation (76) and this can lead to a more pronounced sensitization (77). To investigate the impact of the EcN-Chim and EcN-Ctrl on barrier functions, we evaluated ZO-1 levels in lung tissue samples. The ZO-1 is the protein expressed in tight junctions that regulates the epithelial barrier function by preventing the entry of different pathogens and antigens (78). The EcN strain has been used previously in premature and full-term infants to enhance postnatal immune competence against necrotizing enterocolitis by improving gut barrier function (79). In its outer cell membrane, EcN expresses a unique lipopolysaccharide, enabling EcN to exhibit immunomodulating properties without showing immunotoxic effects (27, 80). It was demonstrated that EcN interacts with the intestinal epithelial cells, which fortifies the epithelial cell barrier. It is also suggested that EcN triggers the stimulation of epithelial defensin production, which helps restore the enterocytes’ tight junctions (81). Our current study shows that poly-sensitization in the gnotobiotic mouse model led to reduced ZO-1 levels compared to GF naïve mice. However, the neonatal application of both the EcN-Chim and EcN-Ctrl maintained the ZO-1 levels in the airways after poly-sensitization at a similar level as in GF naïve mice.

In conclusion, we provide evidence that interventions with probiotic bacteria early in life could present a promising prophylactic tool to imprint the immune system toward preventing the development of poly-sensitization. Contrary to the adult setting, where the mucosal tolerance was induced only in the presence of tolerizing allergen (EcN-Chim) (28), the interventions during peri- and neonatal period of life led to allergy prevention in mice with the probiotic strain without expressed allergen (EcN-Ctrl). In other words, it was possible to induce tolerance avoiding a potential sensitization with “a foreign” antigen. Therefore, we suggest that the application of wild type probiotic bacteria during the “window of opportunity” is a safe approach. Our work builds the foundation for development of personalized strategies for a prophylactic and therapeutic treatment of poly-sensitization. Further studies in mice and humans are needed to evaluate the full potential of our approach.

## Data Availability Statement

The original contributions presented in the study are included in the article. Further inquiries can be directed to the corresponding authors.

## Ethics Statement

The animal study was reviewed and approved by the Animal Experimentation Committee of the Medical University of Vienna and by the Federal Ministry of Science and Research (BMWF-66.009/0384-WF/V/3b/2015) (conventional mice experiments). Gnotobiotic mouse experiments were approved by committee for the Protection and Use of Experimental Animals of the Institute of Microbiology, Academy of Sciences of the Czech Republic (approval ID: 23/2018).

## Author Contributions

UW received the grant from FWF, and together with HK conceived the study. PS, DS, UW designed the experiments. PS, DS, and NG performed the experiments. PS, NG, DS, AI-K, and UW analyzed the data. MS, AI-K, IS, and UW contributed reagents/materials/analysis tools. PS, AI-K, UW drafted the manuscript. PS, AI-K, IS, and UW wrote the manuscript. All authors contributed to the article and approved the submitted version.

## Funding

This work was funded by the Austrian Science Fund (FWF) SFB F46 and DK MCCA W1248-B30; the OeAD WTZ grants CZ 06/2020 and CZ 16/2019; and by the grants: 19-02261S of the Czech Science Foundation and 8J20AT011 of the Ministry of Education, Youth and Sports of the Czech Republic.

## Conflict of Interest

The authors declare that the research was conducted in the absence of any commercial or financial relationships that could be construed as a potential conflict of interest.
